# The Landscape of Wearable Sensors and Automated Literature Analysis with Large-Language Models

**DOI:** 10.1021/acsomega.5c04542

**Published:** 2025-09-10

**Authors:** Jaromir Klarák, Vitor H. B. D Santi, Luan F. Moreira, Robert Andok, Maria Bardosova, Maria Cristina F. Oliveira, Osvaldo N. Oliveira

**Affiliations:** † Institute of Informatics, 112417 Slovak Academy of Sciences, Bratislava 845 07, Slovak Republic; ‡ Sao Carlos Institute of Physics, 42512 University of Sao Paulo, São Carlos 13566-590, Brazil; § Institute of Mathematical Sciences and Computing, University of Sao Paulo, São Carlos, Sao Paulo 13566-590, Brazil

## Abstract

The rapid growth of scientific literature demands advanced methodologies to analyze and synthesize research trends efficiently. This paper explores the integration of complex network analysis and large language models (LLMs) to automate the generation of literature analyses, focusing on the field of wearable sensors for health monitoring. Using OpenAlex as a source of scientific papers in this field, paper citation networks were constructed and partitioned into thematic clusters, revealing key subtopics such as flexible graphene-based sensors, gait analysis, and machine learning applications. These clusters, characterized by their term importance and interconnectivity, served as input for LLMs (ChatGPT) to generate structured outlines and descriptive summaries. While LLMs produced coherent overviews, limitations emerged, including superficial analyses and inaccuracies in referenced literature. The study demonstrates the potential of combining network-based methodologies with LLMs to create scalable literature reviews, albeit with limitations to be addressed concerning depth and accuracy. The analyses highlight wearable sensors’ transformative role in healthcare, driven by advancements in materials science, artificial intelligence, and device integration, while also identifying critical gaps such as standardization, biocompatibility, and energy efficiency. This hybrid approach offers a promising framework for accelerating scholarly synthesis, though today human oversight remains essential to ensure rigor and relevance.

## Graphical abstract

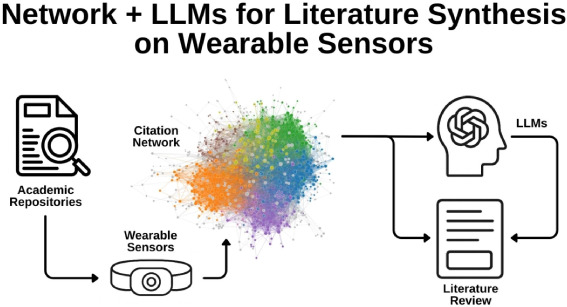

## 1 Introduction

Generative artificial intelligence (AI) based on large language models (LLMs) has the potential to transform academic publishing, including the possibility of machine-generated knowledge, e.g., with AI agents capable of writing scientific papers.^1^ While this applies to various types of publications, surveys and review papers are likely to be the most immediately affected. Literature surveys are essential for scientific research, aiding topic selection, data analysis, and interpretation. Indeed, many journals are dedicated specifically to publishing reviews that provide a broad overview of a given research area. In the past, such an overview could be obtained by consulting a limited number of journals focused on specific topics. However, this is no longer feasible due to the substantial increase in both the number of journals and published articles, as well as the growing multidisciplinary nature of research.^2^ Various techniques now address the vast volume of scientific literature,^3^ primarily through statistical and computational analyses combined with natural language processing (NLP).^4^ In the latter work, for example, the authors retrieved 4,712 arXiv articles using the query “natural language processing,” visualized their relationships with NetworkX^5^ and igraph,^6^ and analyzed problem occurrences across the papers. A study on the use of large language models (LLMs) processed 3,785 studies from PubMed, Scopus, Dimensions, and Google Scholar; from these, 172 were selected for in-depth analysis, covering which stages of the review are automated, which LLM types are proposed for automation, the metrics used to evaluate LLMs, and related factors.^7^

Complex network analysis^8,9^ and machine learning^10^ have also been used. Complex networks are graphs with nontrivial topological characteristics—features absent in simple networks like lattices or random graphs. They employ^11,12^ logical connections between papers, authors, and entities, enabling the identification of communities (i.e., clusters of densely connected elements) through their relationships. A citation network-based methodology was developed to analyze research areas and scientific journal content^13^ and later applied to describe key topics in chemistry and materials science journals.^14^ A similar work to the present study is a review paper focused on the use of graph neural networks in soft sensor development, fault diagnosis, and process monitoring.^15^ An additional advantage of network-based methods is their visualization potential through tools such as Helios-web,^16,17^ VOSviewer,^18,19^ and Gephi,^20^ which are helpful to support exploratory analysis. Many features based on LLMs and NLP are available in these tools—especially in VOSviewer, which is likely the most widely used. For example, social network analysis (SNA) has been conducted on 4,487 Scopus records.^21^ A hydrology-focused bibliometric mapping study used 45 research documents.^22^ A bibliometric analysis of NLP with VOSviewer included 4,803 records,^23^ mapping countries, institutions, authors, and keyword co-occurrence. Another study on agricultural drought analyzed 7,416 studies in VOSviewer.^24^ In summary, with standard tools, most studies operate at the scale of thousands of records (rarely tens of thousands); analyses of 100,000+ papers are very rare.

The analysis of the literature with complex network methodologies can now be complemented with generative artificial intelligences, particularly those based on large language models (LLMs)^25^ such as ChatGPT, Google Gemini, Windows Copilot, and LLaMA (Large Language Model Meta AI), which are capable of generating fluent and contextually relevant texts. This combination has been explored in determining the main materials and methods involved in dressings for wound healing.^26^ However, LLM tools have significant limitations. They tend to produce superficial analyses of complex topics and struggle to provide comprehensive views of highly specific areas. They are not effective when deep understanding of context is required. In this paper we propose a system capable of addressing some of these limitations toward automatically generating literature review articles for a given research topic. Our main contribution is to demonstrate—through a proof-of-concept example—that results from network analysis can be used as input to LLMs to generate a landscape of a given topic. The scientific topic chosen is related to wearable sensors and health monitoring. This field is relevant to many research areas, particularly materials and health, and the devices created are applicable to the Internet of Things and Artificial Intelligence. It is a fast-growing field as indicated in the analysis of the key areas in journals dedicated to applied materials.^14^ We also emphasize the limitations of the use of LLMs for dealing with the scientific literature. In fact, to address these limitations, we conducted this study to demonstrate that combining LLM-based text processing with an interactive web application (**Helios-web**) enables readers to visualize and explore large corpora of papers and automatically generate summaries for specific research areas. The results provide a practical way to cope with the rapid growth in scientific publications and research activity, supporting scalable analysis of large literature data sets. The outline of this paper is as follows. The methodology based on complex networks to yield a landscape of the field is presented in Section 2, along with the description of the system to generate surveys (review papers) autonomously. The landscape for wearable sensors is described in Section 3, while Section 4 discusses texts and analyses generated with LLMs. These texts are included as Supporting Information. Section 5 closes the paper with conclusions and perspectives, especially commenting on the present limitations of LLMs for machine-generated knowledge.

## 2 Methodology

The problem was addressed in a segmented manner, beginning with the retrieval of articles related to the chosen topic, and then the application of the method introduced in ref 13, responsible for generating citation networks and partitioning them into communities. After these steps, LLM tools were used to generate the paper outline and then review articles on the topic of interest, namely “wearable sensors for health monitoring”.

### 2.1 Searches on OpenAlex

The selected topic “wearable sensors for health monitoring” has broad relevance to multiple research areas, such as materials and health. This area features a substantial number of articles, aligning with the goals of this paper and the chosen methodology. The search platform selected for this work was OpenAlex,^27,28^ an open and free database providing information on academic publications, including journals, books and conference papers. OpenAlex offers an API and an interface that facilitates the extraction and use of academic data and citation analysis, along with search filters.^29^ Multiple searches were performed, varying term formulations and search operators (AND, OR, NOT) to maximize the set of articles related to the topic. All searches were conducted with a title and abstract filter (“title and abstract”), as only the contents from these sections of the articles were considered in the subsequent step of network generation.

### 2.2 Network Generation Using the Method from ref 13

The method introduced in ref 13 involves building a citation network from a corpus of scientific articles and applying a community detection algorithm to partition the network into clusters of densely connected articles. The network nodes are formed by the articles, and an edge is established between two articles if one cites the other. Notice that only articles cited by others are included in the network, thus not all retrieved articles will be part of the network, as it will be evident in the results section. Since the goal is to provide a general overview of the scientific topic, analyzing only the most impactful and relevant works within the network is acceptable. For purposes of community detection, we consider the network is undirected. The Louvain method^30^ is applied to partition the network into clusters, which is a stochastic method that produces similar partitions across different runs. Clusters are then characterized by extracting relevant terms—including keywords, unigrams, and bigrams—from the article’s abstracts postprocessed using the downloaded LLM model KeyBERT.^31,32^ Thus, clusters will be characterized by topics and their importance to the field.

A preprocessing step is necessary to remove terms with low semantic content and lemmatize words sharing the same canonical form. The importance of each term within the citation network is quantified by an index that calculates its relative frequency within its community compared to the rest of the network. To determine the frequency of a word (*w*) in a community (α), the total occurrences *n*_α_(*w*) of (*w*) within that community are counted. Then, the relative frequency within the community $F_{\alpha }^{in}\left(\omega \right)$ is given by eq 1:

1 $$F_{\alpha }^{\mathrm{in}}\left(w\right)=\frac{n_{\alpha }\left(w\right)}{\left|\alpha \right|}$$

where |α| is the number of articles within the community (α). Simultaneously, the relative frequency outside the community $F_{\alpha }^{out}\left(w\right)$ is calculated as:

2 $$F_{\alpha }^{out}\left(w\right)=\underset{\gamma \neq \alpha }{\sum }\frac{n_{\gamma }\left(w\right)}{N-\left|\alpha \right|}$$

Here, *N* is the total number of articles in the network. With the internal and external frequency relationships established, the importance *I*(*w*) defined in eq 3 of a word is quantified as the maximum difference between $F_{\alpha }^{in}\left(w\right)$ and $F_{\alpha }^{out}\left(w\right)$ :

3 $$I\left(w\right)=max_{\alpha }\left[F_{\alpha }^{in}\left(w\right)-F_{\alpha }^{out}\left(w\right)\right]$$

In the study from ref 13, keywords ranked by their importance indices are used to create a hierarchical tree that simulates the structure of a survey, generated using an agglomerative hierarchical clustering method.^33,34^ Articles are grouped into general topics, divided into sections and subsections. Keywords are grouped based on the average topological distance between articles containing them, using the average shortest path length $\langle l\rangle_{uv}$ defined in eq 4 between keyword pairs (*u*,*v*):

4 $$\langle l\rangle_{uv}=\underset{\left(u,v\right)\in \left(A_{i}\times A_{j}\right)}{\sum }\frac{l_{ij}}{\left|\left(u,v\right)\in \left(A_{i}\times A_{j}\right)\right|}$$

where *A_i_* and *A_j_* are abstracts of articles where keywords (*u*) and (*v*) are present, respectively, and (*l_ij_*) is the shortest path between article pairs (*i*) and (*j*). This approach groups keywords based on their average topological distance. To address redundancy, unigrams are removed from the keyword set if they are part of a bigram with high *I*(*w*), prioritizing more specific keywords (bigrams). The final output of the method is a cluster-based grouping of words, providing insights into the proximity of concepts within the citation network.

### 2.3 Use of LLM Tools to Generate Outlines and Articles

Large Language Model (LLM) tools can be employed in scientific research, for example, to summarize or describe the concepts covered in provided articles. One of the objectives of this paper is to inform the landscapes obtained using the method from ref 13 to an LLM, prompting the model to produce analyses of the literature and a proof-of-concept review article based on this information. To achieve this, simply providing the network and cluster data to an LLM is insufficient. As previously discussed, LLMs have limitations, such as their tendency to offer superficial analyses on complex topics and difficulty in providing comprehensive views of specific fields. To mitigate these limitations, several strategies were implemented. Multiple LLM tools (e.g., ChatGPT^35^ and Google Gemini^36^) were used to obtain slightly different analyses due to their distinct training methods and algorithms. Text generation was structured to provide input data, such as the cluster tokens with their respective importance indices and the desired textual structure for the review article. LLMs have constraints on text volume; their responses are usually not extensive. To address this, texts were generated segmentally, focusing on one topic at a time. Another issue encountered was related to references. Since we are dealing with a review article, generating content without proper scientific backing would be inappropriate. To resolve this, the tools were instructed to include references in their responses.

## 3 Landscape of the Field “Wearable Sensors for Health Monitoring”

One of the initial challenges in obtaining an overview of a research field is determining its scope. While this might seem straightforward by looking at the number of articles returned from keyword searches, the estimates are often imprecise due to dependence on the search terms used. For example, in the field of interest here—wearable sensors—a simple search using terms like “wearable sensor” or “wearable or sensors” could be unlikely to capture all relevant articles. This occurs because variations, such as “sensing,” may not be included, and articles may discuss wearable sensors without explicitly using those terms. Using more generic searches like “sensor” or “sensing” combined with “wearable” or “wear” might yield a vast number of articles, many of which are irrelevant. Recent experiences using citation networks with the method from ref 13 suggest a potential solution for estimating the scope of the field. This strategy was employed here, using multiple searches to establish upper and lower bounds for the number of articles within the chosen topic. We performed several searches in the OpenAlex database to gather as many articles as possible related to wearable sensors and health monitoring.

The broadest search aimed to investigate the fields of wearable sensors and health monitoring without specifying whether it was for human or animal health, nor requiring that monitoring be conducted with wearable sensors or that wearable sensors be used in health contexts. Using the terms “wearable sensors or health monitoring,” the search returned almost 90,000 articles, of which around half were part of the giant component of the citation network (i.e., the largest subnetwork with no disconnected articles). Due to the broad scope, the clusters identified were highly diverse, with the largest cluster pertaining to the health of bridges, structures, and civil constructions. Therefore, many of the papers retrieved are not related to “human” health monitoring. More restrictive searches, using e.g., “wearable health monitoring” and “wearable medical devices”, retrieved a much smaller number of articles (<20,000), certainly missing relevant content. In these empirical attempts, we found that using the terms “wearable sensors” seemed the best option. It led to over 60,000 papers being retrieved, of which 36,021 belong to the giant component of the citation network (see below). Hence, we may conclude that the upper limit for the number of articles in the OpenAlex database is approximately 60,000. Contrary to the assumption that such a simple search would exclude many relevant articles, this does not seem to have occurred.

Figure 1 shows the citation network containing 36,021 nodes (articles) retrieved with the query “wearable sensors”. The 10 most relevant clusters are indicated in Table 1, along their size in terms of number of papers and the titles created with ChatGPT *4o-mini-high* ^37^ using the OpenAI API. This search did not specify whether the sensors were used for health monitoring. Yet almost all clusters relate to health monitoring. The network is better visualized in the Helios-Web platform, accessible at (http://server1.phys.eu:8080/docs/example/index.html?network=BR_vol2/wearable_sensors_top10_v5B). In another possible visualization, the clusters are deliberately separated from each other to facilitate inspection of individual clusters. This is presented in (http://server1.phys.eu:8080/docs/example/index.html?network=BR_vol2/wearable_sensors_top10_v5). The largest cluster, A (in blue, with 13,986 articles), focuses on flexible and stretchable pressure and strain sensors, which are made mostly with graphene and other nanomaterials, in addition to polymer hydrogels. The second largest cluster, B (in orange, with 12,121 articles), relates to sensors for monitoring human activities, such as gait. In contrast to cluster A, which emphasizes materials for sensors, in Cluster B the major focus is on monitoring technologies. This may explain why Clusters A and B appear at opposite sides in the 3D visualization. The other 8 clusters are much smaller in size, with Cluster C possessing 2,408 papers. Cluster C (in green), like Cluster B, is mostly related to monitoring technology. Cluster D (in red) is related to monitoring with neural networks and other machine learning methods. The monitoring of stress, depression and mental health using wearables is the topic of Cluster E (purple). Cluster F (brown) is associated with data security and communication, while Cluster J focuses on glucose monitoring and diabetes. Cluster G (pink) is completely detached from the other clusters, which is explainable because it is related to monitoring the “health” of tools and industrial processes and materials.

**Figure 1:**
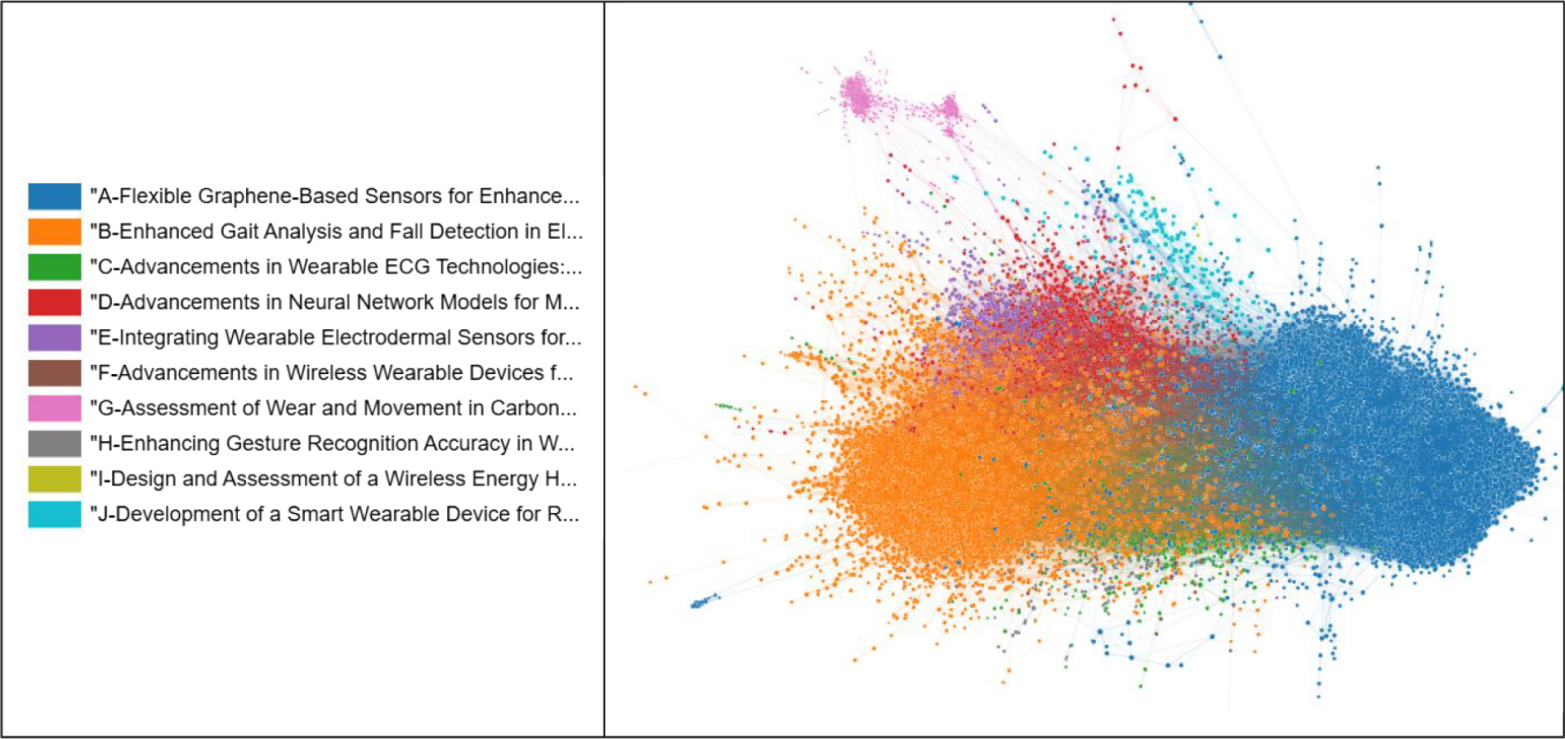
Citation network with 36,021 articles and 257,646 citation connections obtained from the corpus retrieved using the search term “wearable sensors”.

**Table 1:** List of Clusters along with Their Titles and Size (Number of Papers)^a^

**Generated title**	**Cluster size**
A- Flexible Graphene-Based Sensors for Enhanced Temperature and Strain Sensing in Wearable Electronics	13,986
B- Enhanced Gait Analysis and Fall Detection in Elderly Patients Using Wearable Inertial Sensors for Accurate Activity Recognition	12,121
C- Advancements in Wearable ECG Technologies: A Study on Textile-Based Electrodes for Enhanced Health Monitoring and Signal Classification	2,408
D- Advancements in Neural Network Models for Monitoring Physiological Signals: A Comprehensive Study on Energy-Efficient Wearable Devices for Heart Rate Detection	1,940
E- Integrating Wearable Electrodermal Sensors for Real-Time Detection of Stress and Health Monitoring: A Study on Physiological Signals and Heart Rate Variability	1,604
F- Advancements in Wireless Wearable Devices for Healthcare: Integrating IoT Technology for Real-Time Health Monitoring and Data Communication	1,596
G- Assessment of Wear and Movement in Carbon-Based Flexible Films for Real-Time Health Monitoring and Rehabilitation Applications	1,182
H- Enhancing Gesture Recognition Accuracy in Wearable Devices Through Machine Learning and Inertial Data Analysis	747
I- Design and Assessment of a Wireless Energy Harvesting Prototype for IoT Applications Using Carbon Nanotubes and Advanced Signal Processing Techniques	229
J- Development of a Smart Wearable Device for Remote Monitoring of Diabetes Patients Using ECG and Accelerometer Technologies	208

a Titles generated by ChapGPT with version gpt-4o-mini via OpenAI API, with prompt: “Generate only one best title for scientific papers with keywords including importance values:”.

## 4 Literature Analysis Using LLMs

The methodology described can be applied to produce analysis and review articles for any area of knowledge. The flowchart in Figure 2 outlines the procedures for generating a review article in a semiautomated manner by combining the method from ref 13 with subsequent use of LLM tools. The first three steps in the flowchart consist in obtaining the citation network for a topic of interest via the method of ref 13. The resulting network can then be reviewed by the expert (human) requesting the review article. Highlighted in red are the types of information from the networks that can be utilized by LLM tools, including:

List of communities (clusters): These represent the main subtopics within the field, defined by sets of terms whose relevance can be calculated.
List of articles represented by nodes: These articles can be classified based on their centrality in the network. Centrality measures may include simple metrics like the connectivity degree (number of citations an article has received) or more complex metrics like the number of shortest paths passing through the node.
Interconnections between communities and nodes: Relative distances between nodes (articles) are defined, which can provide insight into their relationships.
List of authors, journals, and institutions: This information can aid in analysis.

**Figure 2:**
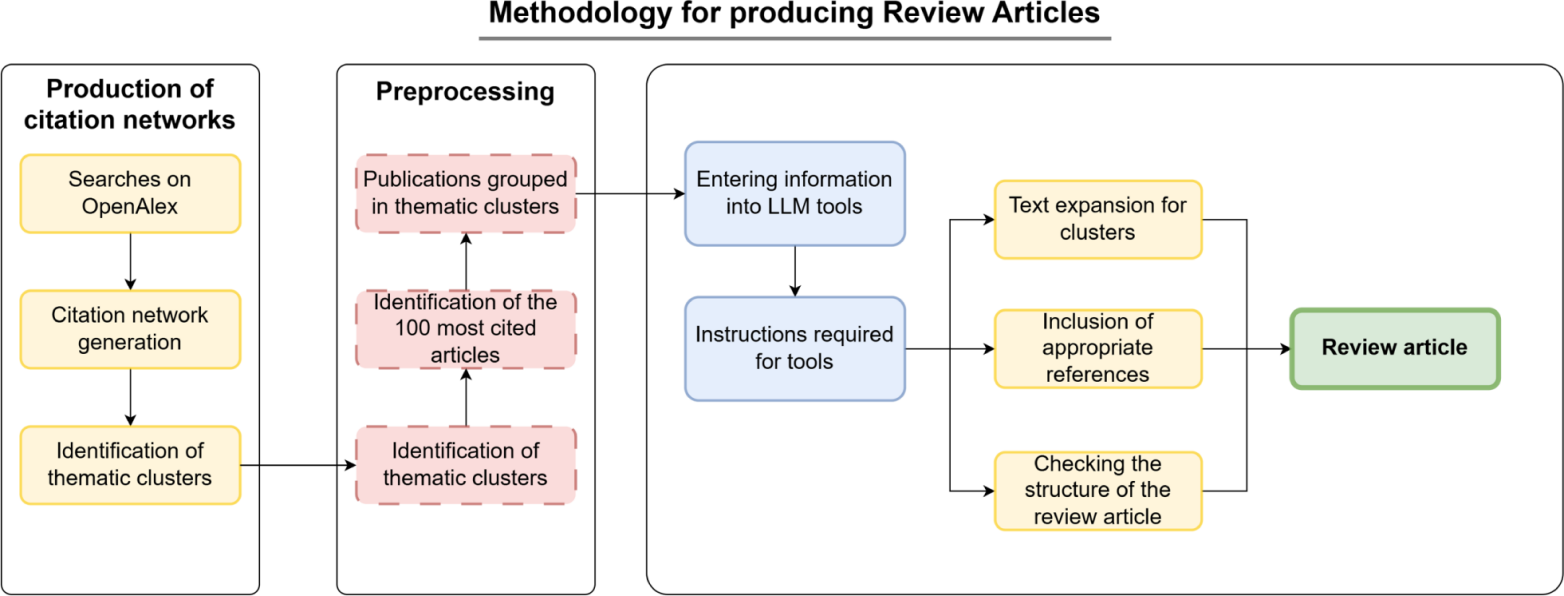
Flowchart for generating review articles using LLMs and thematic citation networks. The pipeline comprises three major blocks. On the left handside are the procedures to obtain a citation network with papers retrieved from a given search in a database such as OpenAlex. The preprocessing steps correspond to partitioning the network into clusters (communities) to identify topics and the representative papers in each topic. The procedures to produce review articles shown on the right involve the use of LLM tools.

We prompted the ChatGPT-*o1* model to generate an outline for a review paper on wearable sensors considering information extracted from the citation network of Figure 1, which identified 10 major clusters of topics in the field. The network information was input by uploading the corresponding .*xnet* file (as described in the Supporting Information). Three other types of information were provided to ChatGPT: i) the cluster sizes, in terms of number of papers in each one; ii) the list of 100 keyterms most representative of each cluster; iii) the importance value of each keyterm. Box 1 shows the outline produced by ChatGPT, with the two first sections expanded to indicate the contents suggested. For Section 3, in particular, specific contents were suggested for each cluster, as can be seen in the complete outline in the Supporting Information. While the entire outline may resemble a possible outline produced by human experts, it is based on a very broad perspective of the field. For it relied on a massive number of papers, rather than on tens or hundreds of papers which could be handled by humans.

It is also true that the contents of the outline generated by ChatGPT are rather generic; therefore, one could argue that similar outlines could be produced simply by employing important keywords from the field. We also asked ChatGPT to provide detailed descriptions of each cluster, using as input the .*xnet* file for the network in Figure 1, the cluster titles, and 100 keywords with their corresponding importance values for each cluster. These descriptions, generated with ChatGPT *o3-mini-high*,^38^ are presented in the Supporting Information. Each cluster is typically described in a two-page text, possibly including subsections and ending with a list of key references. Although these descriptions are brief and do not report specific results from the literature, together they offer a broad overview of the field. Some of the references introduced by ChatGPT *o3-mini-high* include incorrect or nonexistent entries. This limitation can potentially be addressed by incorporating additional tools or strategies to ensure that all references exist in a validated database such as OpenAlex.

Even without details on specific results, the cluster descriptions in the Supporting Information span over 30 pages. Since one of the objectives of our paper is to provide an analysis of the field—though without the intention of producing a fully fledged review—we asked ChatGPT-4o^39^ to generate a 1,000-word summary of the clusters content. The texts obtained from this description of the clusters were quite informative. They were edited to produce the discussion below:

The cluster analysis clearly shows that wearable sensors are transforming healthcare by enabling continuous, real-time monitoring of physiological and biomechanical signals. These devices are designed to be lightweight, flexible, and comfortable for long-term wear, making them ideal for applications such as chronic disease management, rehabilitation, and early detection of health anomalies. Progress in this field has been driven by advances in materials science, sensor design, signal processing, and wireless connectivity, all aimed at improving the accuracy, usability, and clinical relevance of wearable health technologies. The sensing modalities discussed primarily include flexible and stretchable sensors made from materials such as graphene, carbon nanotubes (CNTs), and conductive polymers, often embedded in hydrogels or textiles. Other device types include accelerometers and gyroscopes for monitoring movement, as well as skin-adhered sensors for detecting heart rate, stress levels, and other physiological conditions. It is worth noting that the summaries produced by the LLM tools did not mention the integration of wearable sensors with biosensors. This omission is likely due to the relatively smaller number of publications on wearable biosensors, despite their importance for health monitoring. In fact, there is a significant imbalance in the literature when comparing biosensors and physical sensors. Wearable biosensors are underrepresented, a trend that is reflected in the landscape analysis presented in our work.

Regarding the techniques used to produce wearable sensors, additive manufacturing is prominently featured, with particular emphasis on methods such as inkjet printing, screen printing, and 3D printing, as well as laser patterning for the precise fabrication of conductive traces on flexible substrates. Since powering the sensors is a critical challenge, nanogenerators and energy-harvesting strategies have been employed in self-powered devices. In terms of applications, particular emphasis is placed on the management of chronic diseases, such as diabetes and cardiovascular conditions, rehabilitation through physical condition monitoring, mental health and stress tracking, and elderly care. The growing use of machine learning and other AI methods is also reflected in some of the clusters, as effective health monitoring relies on advanced signal processing and data analysis. Several feature extraction and classification algorithms were identified, primarily based on machine learning, including deep learning approaches. These methods enable so-called multimodal fusion, which combines data from multiple sensors to improve the accuracy of health assessments. This is closely linked to wireless communication and Internet-of-Things (IoT) integration, allowing for continuous monitoring and real-time analysis. The brief description of the use of machine learning for processing data from wearable sensors clearly demonstrated the potential of this field—especially given that future health monitoring systems will likely rely entirely on the integration of machine learning and wearable devices.

As with any literature analysis, the summary produced by the LLM tools includes a discussion of the challenges and prospects of the field. For sensors, in particular, there is an ongoing effort to achieve long-term stability and biocompatibility. To this end, hypoallergenic materials and encapsulation techniques are being developed to improve compatibility with the human body. Another major challenge is the lack of standardized protocols for data collection and processing, which hinders cross-study comparisons. Energy efficiency is also a critical issue, with energy-harvesting technologies and ultralow-power designs being essential for enabling long-term operation. Also highlighted is the concept of personalized medicine, with increasing reliance on AI methodologies to tailor healthcare to individual needs. Finally, critical barriers to the widespread adoption of wearable sensors in medical applications include the need for clinical validation and regulatory compliance, along with ethical considerations that must be carefully addressed.

The analysis above does not allow one to determine whether an intelligent system could provide an in-depth, authoritative discussion of the field. In order to test this, we used ChatGPT to write a short review paper on Cluster A from Figure 1, including figures and references. The results can be seen in the Supporting Information. The title chosen by ChatGPT for the review paper was “Advances and Trends in Flexible and Wearable Sensor Technologies: A Network-Based Review”, which is excellent for conveying the intended focus, also emphasizing the distinct nature of the review, based on network analysis.

The automatically generated review is an excellent starting point for a paper on wearable sensors made with graphene and other carbon materials. However, the strong focus on graphene may not be ideal, as Cluster A is broad and includes other relevant materials that should also be addressed. Nevertheless, the text does establish connections between graphene and these other materials. The abstract and outline of the generated paper are excellent, as the main topics are well covered. In fact, the review provides a comprehensive overview of flexible, graphene-based temperature and strain sensors for wearable electronics, detailing material innovations, fabrication strategies, sensing mechanisms, performance benchmarks, integration approaches, and key challenges. The significance of graphene in the field is justified by its unique properties.

The major topics are organized into eight distinct sections that discuss materials and fabrication techniques, sensing mechanisms, performance metrics, integration into wearable devices, applications, challenges, and future perspectives. While the selection of topics and the overall text are generally appropriate, there are notable stylistic and content-related shortcomings. First, the excessive use of itemization disrupts the text flow. More importantly, the descriptions are mostly superficial—an issue commonly found in texts generated by large language models. This superficiality is arguably the greatest hurdle in producing review papers suitable for prestigious journals. Another limitation is the small number of figures, all of which were generated by the LLM tool. Although these illustrations are appropriate, a high-quality review should ideally include several figures from published literature. A dedicated tool will be needed to address this limitation effectively.

## 5 Conclusions, Limitations, and Perspectives

This study presents an analysis of the field of wearable sensors for health monitoring using a hybrid approach to automate literature reviews by combining complex network analysis with large language models (LLMs). By constructing citation networks from OpenAlex data and applying clustering algorithms, we identified major subtopics in the field and used LLMs to generate structured summaries and review drafts. This methodology offers a scalable framework for navigating vast scientific corpora and provides insights into the thematic structure and evolution of interdisciplinary domains. The main findings indicate that progress in wearable sensors has been largely driven by advances in materials science—particularly related to carbon materials and polymers—as well as in device engineering, including the development of self-powered devices. Combined with the use of machine learning and other AI methodologies, these wearable sensors are increasingly applied to various aspects of health monitoring, such as chronic disease management and rehabilitation. The main contribution of our work lies in demonstrating that network analysis can be effectively combined with LLM tools to generate surveys on any given topic. Furthermore, the pipeline used for generating these surveys can be readily adopted by other authors to implement their own systems for machine-generated reviews and surveys.

Our analysis also highlights both the potential and current limitations of LLMs in scientific synthesis. While the generated texts were coherent and thematically relevant, they often lacked depth and included superficial analyses, particularly when describing technical details or contextualizing findings. Furthermore, the inclusion of inaccurate or fabricated references highlights the need for robust validation pipelines and integration with curated databases. These issues confirm that, at present, human oversight remains essential to ensure the accuracy, rigor, and interpretability of machine-generated content. Several limitations of this study must also be acknowledged. First, while the citation network methodology delineates thematic clusters, its performance depends on search term selection and database coverage, which may result in the omission of relevant subfields. Second, although multiple LLMs were used to reduce bias, they still struggle with context-dependent understanding and may reproduce errors or hallucinations. We also observed that the ChatGPT *o3-mini-high* model hallucinated references in the Supporting Information. To prevent further LLM hallucinations, we verified the 100 top-cited references (listed at the end of the Supporting Information) using OpenAI’s Scholar GPT. LLMs are trained on data up to a fixed cutoff and can be biased toward more frequently occurring information—often older sources from three to five years ago—while underrepresenting newer findings. For this reason, human judgment remains essential to ensure the appropriateness and accuracy of content generated by LLMs. That is to say, domain expertise is still essential to interpret and polish the LLM-generated content. Third, the lack of figures derived from validated sources and the minimal incorporation of actual article content into the summaries are notable shortcomings when compared to conventional review articles. Another limitation is that we generated content using only keywords extracted from abstracts. Deeper insights could be achieved by analyzing the full text of the papers, as in a recent study.^40^ The reproducibility is also limited due to the “non-deterministic” nature of LLMs, which are updated constantly.

Despite these constraints, the approach opens new perspectives for accelerating scientific synthesis, especially in fast-growing or highly multidisciplinary fields. Future developments should focus on improving LLM accuracy through the incorporation of structured metadata, reference validation tools, and deeper integration with citation contexts. Tools capable of automatically extracting and summarizing quantitative results, figures, and methodological nuances from primary literature would further enhance the quality of reviews. Additionally, establishing community standards for the evaluation of AI-assisted reviews could facilitate broader adoption while preserving academic integrity. While not yet a replacement for expert-driven reviews, the methodology presented here offers a valuable tool for augmenting scholarly work, informing research directions, and democratizing access to scientific overviews. The efficiency of deploying LLM-based methods in review processing can be improved at multiple levels. First, preparing high-quality metadata helps eliminate redundant operations. Second, setting realistic expectations for the outputs and choosing an appropriate model are essential to achieving the desired results.

## Supplementary Material



## Notes

Outline of a review paper generated automatically, description of clusters from networks, and automatically generated review paper for one cluster of the network (PDF)
